# Optic disc Edema in patients with fibrous dysplasia/McCune-Albright syndrome: Craniomorphometric analysis and peripapillary retinal nerve fiber layer data

**DOI:** 10.1016/j.dib.2021.107449

**Published:** 2021-10-03

**Authors:** Layne N. Raborn, Kristen S. Pan, Edmond J. FitzGibbon, Michael T. Collins, Alison M. Boyce

**Affiliations:** a Skeletal Disorders and Mineral Homeostasis Section, National Institute of Dental and Craniofacial Research, National Institutes of Health, Bethesda, MD, USA; b Laboratory of Sensorimotor Research, National Eye Institute, National Institutes of Health, Bethesda, MD, USA

**Keywords:** Papilledema, OSIRIX, Optic canal area, Retinal nerve fiber layer, Intracranial volume, Spectral-domain optical coherence tomography, 3D multiplanar reconstruction, Optic neuropathy

## Abstract

This article reports quantitative measurements of intracranial volume, optic canal area, and peripapillary retinal nerve fiber layer (RNFL) for a cohort of 124 patients with craniofacial fibrous dysplasia/McCune-Albright Syndrome (FD/MAS), previously used to determine risks for developing optic disc edema [1]. Of these, 7 subjects were diagnosed with optic disc edema. OSIRIX imaging analysis software was used to collect intracranial volume and optic canal diameter for 107 patients, via 3D multiplanar reconstruction (MPR) of ≤5 mm axial CT slices. Spectral-domain Optical Coherence Tomography (OCT) was performed with the Cirrus-HD OCT (Carl Zeiss Meditec, Inc., Dublin, CA). The Optic Disc Cube 200 × 200 protocol was used for acquisition and analysis of the RNFL for 69 patients. The data can be used to assess typical ranges for intracranial volume, optic canal area, and RNFL in the craniofacial FD/MAS population and to assess ranges concerning for optic disc edema.

[1] Raborn LN, Pan KS, FitzGibbon EJ, Collins MT, Boyce AM. Optic disc edema in fibrous dysplasia/McCune-Albright syndrome: Prevalence, etiologies, and clinical implications. Bone. 2021 Feb;143:115661. doi: 10.1016/j.bone.2020.115661. Epub 2020 Sep 24. PMID: 32979536.

## Specifications Table

**Table:** 

Subject	Endocrinology, Diabetes and Metabolism
Specific subject area	Fibrous dysplasia/McCune-Albright Syndrome (FD/MAS) affected cohort craniomorphometric and peripapillary nerve fiber layer (RNFL) measurements
Type of data	Table, Figure
How data were acquired	OSIRIX imaging analysis software and Spectral-domain Optical Coherence Tomography (OCT) using Cirrus-HD OCT (Carl Zeiss Meditec, Inc., Dublin, CA)
Data format	Raw
Parameters for data collection	OSIRIX imaging analysis was used with region of interest function to calculate intracranial volume and optic canal area acquired through 3D multiplanar reconstruction of ≤5 mm axial CT slices, Optic Disc Cube 200 × 200 protocol was used for acquisition and analysis of the RNFL
Description of data collection	OSIRIX software was used to determine intracranial volume and optic canal area. Region of interest (ROI) was outlined on CT images with ≤5 mm axial slices. Volume was calculated by adding all ROIs outlining intracranial area. 3D multiplanar reconstruction (MPR) was used to align axial, coronal, and sagittal views of the optic canal and ROI outlining the canal yielded area. Spectral-domain Optical Coherence Tomography (OCT) was performed with the Cirrus-HD OCT. The Optic Disc Cube 200 × 200 protocol was used for acquisition and analysis of the peripapillary retinal nerve fiber layer (RNFL).
Data source location	National Institutes of Health Bethesda, MD USA
Data accessibility	With the article
Related research article	Raborn LN, Pan KS, FitzGibbon EJ, Collins MT, Boyce AM. Optic disc edema in fibrous dysplasia/McCune-Albright syndrome: Prevalence, etiologies, and clinical implications. Bone. 2021 Feb;143:115661. 10.1016/j.bone.2020.115661. Epub 2020 Sep 24. PMID: 32979536.

## Value of the Data

• We demonstrate a reliable method of determining optic canal area and intracranial volume using OSIRIX imaging analysis software and the data we collected by this method for a cohort of patients with FD/MAS and craniofacial involvement. We present RNFL measurements for a range of patients, including 7 diagnosed with optic disc edema and 62 with no optic disc edema for future comparison.
• This data is useful for investigators and clinicians caring for patients with FD/MAS.
• This data can be used to further study the effect of FD/MAS disease severity on intracranial volume and optic canal area or to investigate the utilization of RNFL in predicting optic disc edema.
• FD/MAS is a rare disease, and we provide data from the largest cohort study of optic disc edema to date which can be used for future research.
• The usefulness of OCT in the population is limited by a lack of standard ranges for pediatric patients, which could hinder its usefulness in identifying optic disc edema. We present a large cohort of patients with RNFL data that can be utilized to identify RNFL dimensions concerning for optic disc edema.

## 1 Data Description

Craniomorphometric analysis and peripapillary retinal nerve fiber layer data in patients with craniofacial fibrous dysplasia.

## 2 Experimental Design, Materials and Methods

A full description of study design, methods, participant characterization, and optic disc edema diagnosis can be found in our corresponding published literature [1]. Subjects were evaluated between 2000 and 2019 at the National Institutes of Health as part of an ongoing natural history study of FD/MAS (NCT00001727). The study was approved by the Institutional Review Board of the National Institute of Dental and Craniofacial Research, and informed consent/assent was obtained from all subjects and/or guardians. All subjects were diagnosed with craniofacial FD/MAS according to previously published guidelines [2] and diagnosed with optic disc edema by neuro-ophthalmologic examination [1]. Subject ODE-2 had optic disc edema in the left eye only and optic neuropathy diagnosed in the right eye. Subject ODE-7 was diagnosed with optic disc edema prior to evaluation and was started on Acetazolamide therapy. At the time of evaluation, her optic disc edema was resolved.

**Fig. 1:**
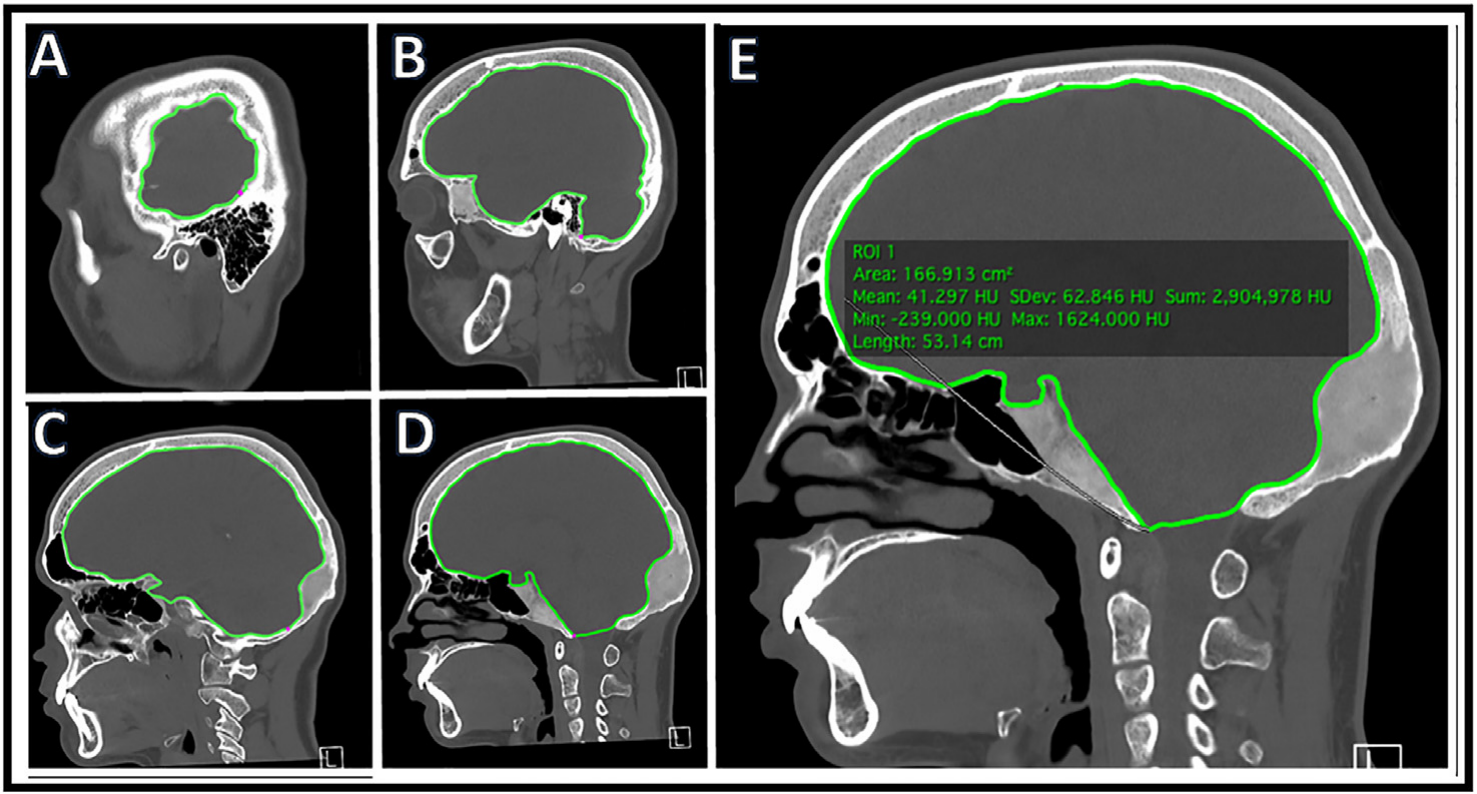
Craniomorphometric analysis of intracranial volume in a subject with fibrous dysplasia/McCune-Albright syndrome, utilizing OSIRIX software. Using CT head imaging with ≤5 mm axial slices, a region of interest (ROI) was traced to include intracranial area. Tracing was started with lateral slices (A, B) and advanced towards medial slices (C-E). The software calculated the area encompassed within ROIs and used this to determine total volume (E).

**Fig. 2:**
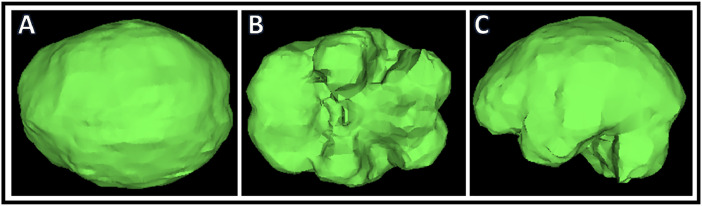
Intracranial volume was determined through a summation of region of interests (ROIs). A 3D rendering of the volume was generated using OSIRIX software. A) Top down B) Bottom up and C) Sagittal views of 3D rendering are shown.

**Fig. 3:**
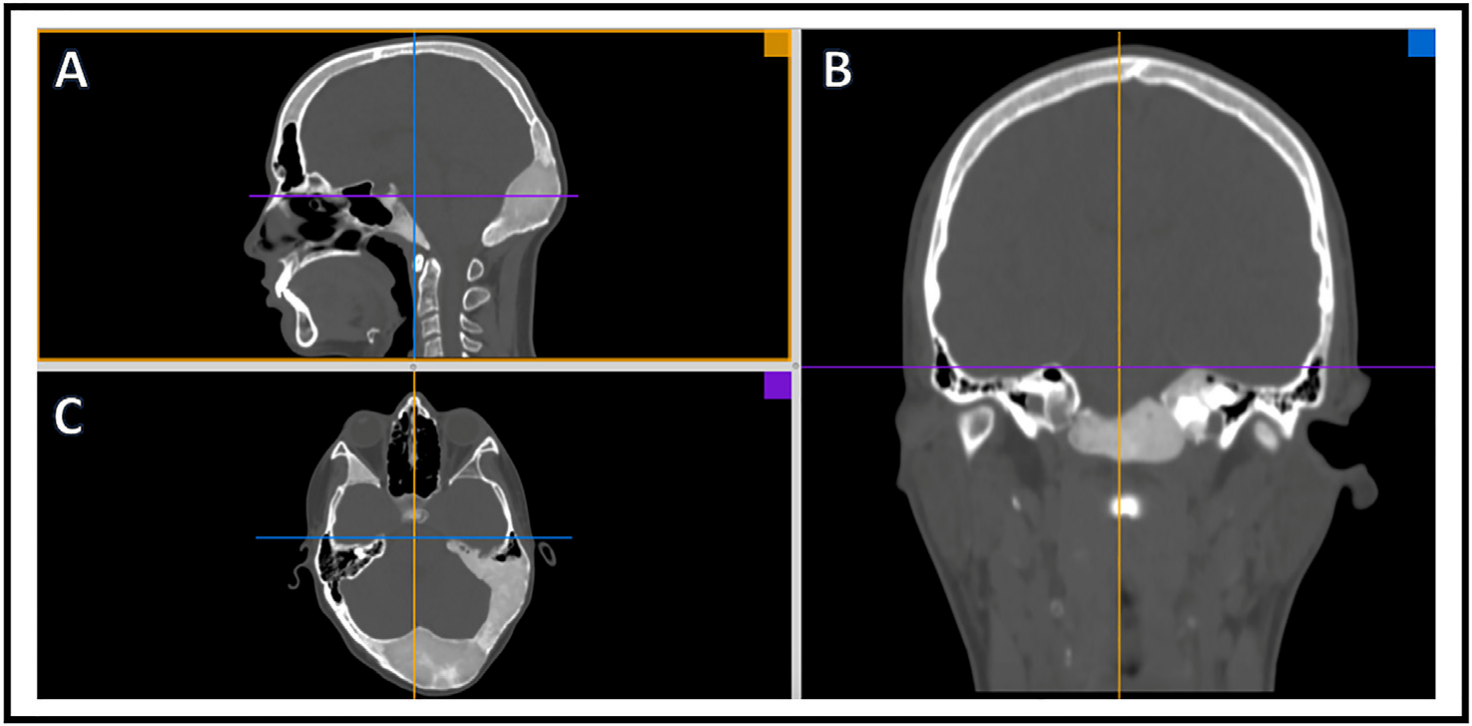
3D Multiplanar Reconstruction with OSIRIX software allowed for simultaneous visualization of sagittal plane (A) in yellow, coronal plane (B) in blue, and axial plane (C) in purple.

**Fig. 4:**
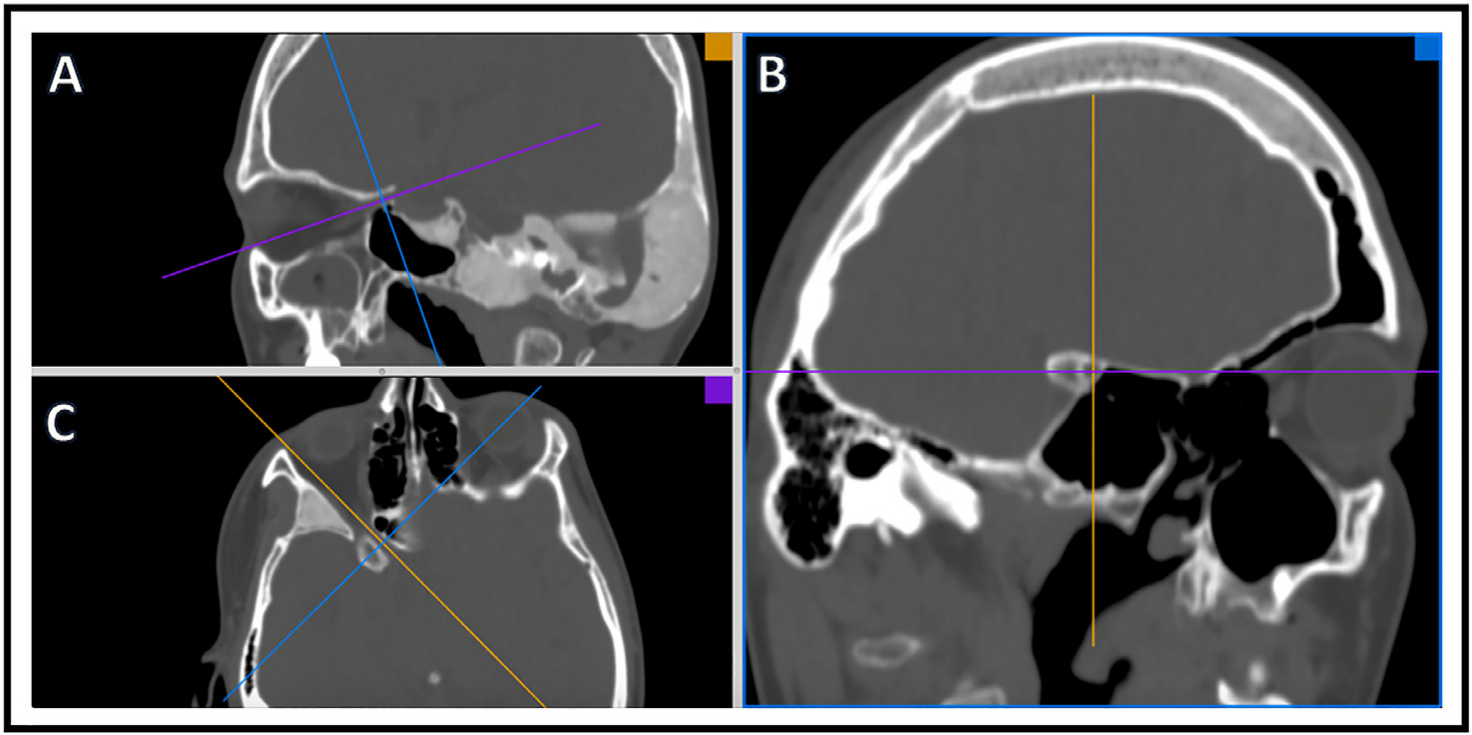
Optic canal was aligned using 3D Multiplanar Reconstruction in a subject with fibrous dysplasia/McCune-Albright syndrome with OSIRIX imaging software in sagittal plane (A) in yellow, coronal plane (B) in blue, and axial plane (C) in purple.

**Fig. 5:**
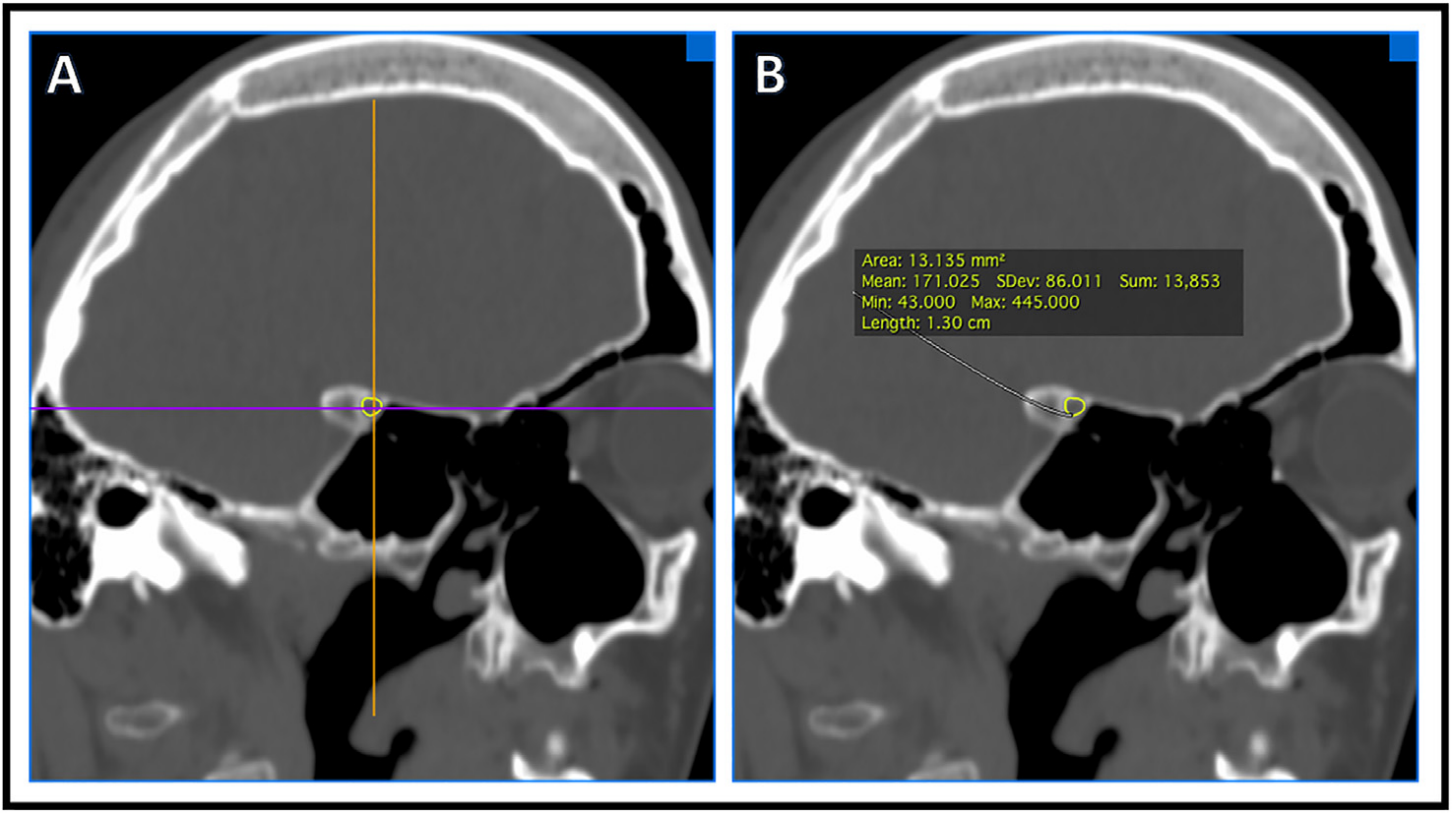
Using coronal plane rendering of optic canal aligned with 3D Multiplanar Reconstruction, the region of interest (ROI) was traced around the optic canal (A) and used to determine area through OSIRIX software (B).

Craniomorphometric analyses was performed using OSIRIX imaging analysis software and a single trained reader (KSP) to determine intracranial volume and optic canal area. All analysis utilized CT head imaging with axial slices ≤5mm. To determine intracranial volume, a region of interest (ROI) was traced manually to include intracranial area only (Fig. 1). All lateral CT slices were manually traced because of variation in intracranial calvarial contour (Fig. 1A, B). Midline slices typically showed less intracranial calvarial contour variation and were manually traced every 3-5 slices (Fig. 1C, D). Using the software, remaining ROIs were automatically generated. The reader examined automated ROI tracings and manually corrected errors. Each ROI contained intracranial area that was used to determine total intracranial volume (Fig. 1E). Using OSIRIX, intracranial volume was calculated from the outlined ROIs (ICV = Σ (A1, A2, . . ., Az)  ×  CT slice thickness). A 3D volume was rendered (Fig. 2) along with volume output and is described in Table 1. Optic canal area was also determined using OSIRIX software and CT head imaging with ≤5 mm axial slices. 3D Multiplanar Reconstruction (MPR) was utilized, which allowed for simultaneous visualization of the optic canal in axial, sagittal, and coronal planes (Fig. 3). The optic canal was then aligned in each plane (Fig. 4). Using coronal plane rendering of optic canal, the ROI function was used to manually trace the optic canal using digital calipers and determine the area (Fig. 5). Optic canal area for each eye is listed in Table 1.

**Table 1:** Craniomorphometric measurements of intracranial volume and optic canal area for craniofacial fibrous dysplasia/McCune-Albright Syndrome cohort.

Subject	Sex	Age (years)	Race/Ethnicity	Intracranial Volume (cm^3^)	OD OCA (mm^2^)	OS OCA (mm^2^)
Control 1	F	19	White	1275	14.02	13.4
Control 2	F	34	White	1289	10.03	11.5
Control 3	F	33	White	1129	11.89	9.1
Control 4	M	19	Black or African American	1298	12.53	12.72
Control 5	F	60	White	1360	13.56	10.78
Control 6	M	41	White	1622	19.21	19.2
Control 7	F	22	White	1456	11.32	11.94
Control 8	F	28	White	1318	16.12	13.67
Control 9	F	21	White	1170	12.76	12.72
Control 10	F	34	White	1309	12.01	12.72
Control 12	M	33	White	1432	15.75	14.66
Control 13	F	28	White	1135	10.34	9.52
Control 14	M	30	White	1523	11.01	11.06
Control 15	F	27	Hispanic or Latino	1155	12.08	12.22
Control 18	M	50	White	1432	16.96	16.67
Control 19	F	18	White	1339	10.91	10.18
Control 20	F	56	White	1491	17.86	17.38
Control 21	M	17	White	1624	18.28	20.25
Control 22	M	57	White	1390	12.51	12.72
Control 23	F	46	Hispanic or Latino	1201	19.41	16.52
Control 24	F	57	White	1299	14.16	12.51
Control 25	F	23	Asian	1306	10.07	11.16
Control 26	F	23	White	1269	12.42	12.88
Control 27	M	22	White	1503	19.63	13.32
Control 29	M	35	Asian	1337	15.28	13.66
Control 30	F	18	Hispanic or Latino	1189	15.16	11.58
Control 31	F	47	White	1266	12.45	12.04
Control 32	F	9	White	1333	11.47	10.93
Control 33	M	15	White	1790	11.81	13.56
Control 34	F	19	White	1436	11.51	12.84
Control 35	M	46	White	1422	14.21	12.59
Control 36	F	13	White	1419	15.95	16.61
Control 37	F	52	White	1359	21.42	18.97
Control 38	M	48	Asian	1419	12.71	12.09
Control 39	F	37	White	1245	11.6	11.06
Control 40	M	11	White	1272	12.37	10.6
Control 41	F	21	Asian	1338	10.62	10.84
Control 42	F	6	White	1306	12.66	11.77
Control 43	M	13	White	1425	10.31	10.17
Control 44	M	16	White	1258	13.59	12.95
Control 45	M	19	White	1414	14.82	14.45
Control 46	F	11	White	1120	8.8	7.57
Control 48	M	13	White	1395	12.64	13.24
Control 49	F	6	White	1029	9.77	8.95
Control 50	M	8	Asian	1414	18.73	17.42
Control 51	M	59	Hispanic or Latino	1559	19.02	18.74
Control 52	F	9	White	1208	10.69	11.38
Control 53	F	22	White	1255	14.52	15.31
Control 54	M	10	Black or African American	1408	12.09	11.06
Control 55	F	24	White	1542	12.85	12.65
Control 56	F	57	White	1205	14.32	13.79
Control 57	M	50	White	1421	15.12	14.36
Control 59	M	10	White	1590	12.1	10.98
Control 61	F	15	Black or African American	1034	11.58	10.66
Control 62	F	5	White	1023	11.96	11.65
Control 63	M	20	Hispanic or Latino	1605	15.8	14.57
Control 64	F	5	White	1395	13.34	13.58
Control 65	M	20	White	1583	17.63	17.74
Control 66	M	24	White	1482	12.37	12.9
Control 69	M	18	White	1475	16.57	15.7
Control 70	F	5	Asian	1399	14.94	10.82
Control 71	F	80	White	1432	11.01	10.03
Control 72	F	6	White	1394	13.39	13.37
Control 74	M	10	White	1410	13.39	14.24
Control 77	F	9	White	1395	15.87	15.79
Control 78	F	10	White	1319	10.4	11.79
Control 79	F	37	White	1273	12.85	13.18
Control 80	F	8	White	1328	19.18	19.52
Control 81	M	8	Hispanic or Latino	1404	18.19	17.77
Control 83	F	35	Asian	1414	13.08	13.25
Control 84	F	7	White	1180	12.73	13.76
Control 85	M	30	White	1358	11.81	11.46
Control 86	F	9	White	1315	10.88	10.95
Control 87	F	16	Multiple Race	1349	7.54	11.65
Control 88	F	6	White	1351	18.55	17.99
Control 89	M	16	Hispanic or Latino	1359	17.86	19.51
Control 91	M	9	White	1287	12.27	11.71
Control 92	M	12	White	1526	15.46	17.45
Control 93	M	4	White	1303	13.38	11.11
Control 94	F	19	White	1452	15.25	13.14
Control 95	M	10	White	1452	13.43	13.64
Control 97	M	25	Multiple Race	1747	12.37	11.25
Control 98	M	52	White	1452	10.49	10.72
Control 99	M	6	Hispanic or Latino	1462	14.25	12.79
Control 100	F	3	Asian	1418	15.78	15.24
Control 101	F	19	White	1330	12.71	13.55
Control 102	M	4	Hispanic or Latino	1240	18.15	14.46
Control 103	F	55	White	1239	18.31	15.78
Control 104	F	26	White	1578	17.25	15.82
Control 105	M	11	White	1388	10.46	8.47
Control 106	F	5	Multiple Race	1210	14.97	14.02
Control 109	F	6	Asian	1304	15.09	15.59
Control 110	M	19	White	1294	11.59	11.8
Control 111	F	32	White	1616	17.7	17.04
Control 112	F	27	White	1223	8.51	9.61
Control 113	F	3	White	997	10.11	11.59
Control 114	F	43	White	1297	9.8	9.46
Control 115	F	6	White	1167	13.06	12.74
Control 116	F	69	White	1397	12.94	10.42
Control 117	M	12	Black or African American	1019	11.9	12.35
ODE 1	M	14	White	1354	11.39	14.96
ODE 2	M	15	White	1316	8.85*	8.61
ODE 3	M	12	White	1611	16.93	12.39
ODE 4	M	17	White	1655	17.75	15.04
ODE 5	F	17	Asian	1224	15.48	12.19
ODE 6	M	7	White	1290	14.46	17.28
ODE 7**	F	5	Asian	1004	12.04	11.26

Both intracranial volume and optic canal area were collected by importing Computed Tomography (CT) images into OSIRIX software, which were then used to create a 3D reconstruction of the image.

Intracranial area and the area of the optic canal were measured by outlining the area of interest within image cross-sections.

Intracranial volume was calculated via OSIRIX software which sums the intracranial area outlined in all cross-sections and multiplies by cross-section thickness.

Race and ethnicity were self-reported by each subject.

ODE = subject with diagnosed optic disc edema, Control = subject with no diagnosis of optic disc edema, M = male, F = female, OD = right eye, OCA = optic canal area, OS = left eye, (*) = subject had diagnosed optic neuropathy in right eye, (**) = subject was diagnosed with ODE prior to visit and showed resolution during time of exam on Acetazolamide therapy.

Spectral-domain Optical Coherence Tomography (OCT) was performed with the Cirrus-HD OCT (Carl Zeiss Meditec, Inc., Dublin, CA). The Optic Disc Cube 200 × 200 protocol was used for acquisition and analysis of the total RNFL and recorded in Table 2.

**Table 2:** Peripapillary nerve fiber layer (RNFL) measurements of craniofacial fibrous dysplasia/McCune-Albright Syndrome cohort.

Subject	Sex	Race/Ethnicity	Age (years)	RNFL OD (µn)	RNFL OS (µn)
Control 3	F	White	33	83	78
Control 7	F	White	22	93	100
Control 8	F	White	28	100	97
Control 11	F	White	32	86	79
Control 12	M	White	33	111	107
Control 14	M	White	30	75	81
Control 15	F	Hispanic or Latino	27	105	107
Control 16	F	White	32	108	109
Control 17	F	Asian	28	109	101
Control 21	M	White	17	102	96
Control 22	M	White	57	80	77
Control 24	F	White	57	106	107
Control 27	M	White	22	93	91
Control 28	M	White	56	79	82
Control 31	F	White	47	94	94
Control 34	F	White	19	101	101
Control 44	M	White	16	96	95
Control 45	M	White	19	90	92
Control 47	M	White	18	94	94
Control 58	F	White	18	105	103
Control 60	F	White	24	100	98
Control 63	M	Hispanic or Latino	20	114	110
Control 65	M	White	20	110	109
Control 66	M	White	24	94	99
Control 67	F	Asian	11	98	106
Control 68	F	Asian	31	90	94
Control 69	M	White	18	89	112
Control 72	F	White	6	98	97
Control 73	F	Asian	11	92	94
Control 74	M	White	10	95	97
Control 75	F	White	20	103	99
Control 76	F	White	8	103	103
Control 77	F	White	9	90	91
Control 78	F	White	10	102	106
Control 80	F	White	8	110	99
Control 82	F	White	49	94	92
Control 83	F	Asian	35	111	112
Control 84	F	White	7	106	112
Control 85	M	White	30	106	101
Control 86	F	White	9	116	110
Control 87	F	Multiple Race	16	106	110
Control 89	M	Hispanic or Latino	16	83	85
Control 90	F	White	8	84	96
Control 91	M	White	9	95	107
Control 93	M	White	4	115	115
Control 94	F	White	19	101	101
Control 95	M	White	10	91	116
Control 96	F	Hispanic or Latino	6	91	89
Control 98	M	White	52	87	90
Control 99	M	Hispanic or Latino	6	105	103
Control 101	F	White	19	96	101
Control 102	M	Hispanic or Latino	4	76	77
Control 103	F	White	55	85	88
Control 105	M	White	11	96	90
Control 107	M	Black or African American	5	107	89
Control 108	M	White	66	88	90
Control 109	F	Asian	6	100	93
Control 110	M	White	19	84	92
Control 111	F	White	32	104	105
Control 112	F	White	27	105	111
Control 115	F	White	6	107	100
Control 117	M	Black or African American	12	129	119
ODE 1	M	White	13	124	96
ODE 2	M	White	15	67*	139
ODE 3	M	White	11	118	158
ODE 4	M	White	17	154	198
ODE 5	F	Asian	16	125	113
ODE 6	M	White	7	139	132
ODE 7**	F	Asian	5	110	101

RNFL measurements were acquired via Spectral-domain Optical Coherence Tomography (OCT), using a Cirrus-HD OCT, and the Optic Disc Cube 200 × 200 protocol.

Race and ethnicity were self-reported by each subject.

ODE = subject with diagnosed optic disc edema on exam, Control = subject with no diagnosis of optic disc edema, M = male, F = female, RNFL = peripapillary nerve fiber layer, OD = right eye, OS = left eye, (*) = subject had diagnosed optic neuropathy in right eye, (**) = subject was diagnosed with ODE prior to visit and showed resolution during time of exam on Acetazolamide therapy.

## Ethics Statement

Subjects were enrolled in the National Institutes of Health ongoing natural history study of FD/MAS (NCT00001727). The study was approved by the Institutional Review Board of the National Institute of Dental and Craniofacial Research, and informed consent/assent was obtained from all subjects and/or guardians.

## CRediT authorship contribution statement

**Layne N. Raborn:** Conceptualization, Methodology, Investigation, Visualization, Writing – original draft, Data curation. **Kristen S. Pan:** Conceptualization, Methodology, Software, Validation, Formal analysis, Data curation, Investigation. **Edmond J. FitzGibbon:** Resources, Writing – review & editing, Supervision, Project administration, Funding acquisition. **Michael T. Collins:** Conceptualization, Resources, Writing – review & editing, Supervision, Project administration, Funding acquisition. **Alison M. Boyce:** Conceptualization, Resources, Writing – review & editing, Supervision, Project administration, Funding acquisition, Supervision, Visualization.

## Declaration of Competing Interest

NIDCR receives funding from Amgen, Inc and Ultragenyx, Inc for studies in fibrous dysplasia.
